# Complete photonic bandgap in silicon nitride slab assisted by effective index difference between polarizations

**DOI:** 10.1007/s12200-022-00023-6

**Published:** 2022-05-06

**Authors:** Can Ma, Jin Hou, Chunyong Yang, Ming Shi, Shaoping Chen

**Affiliations:** Hubei Key Laboratory of Intelligent Wireless Communications, Hubei Engineering Research Center for Intelligent Internet of Things, College of Electronic and Information Engineering, South-Central MinZu University, Wuhan, 430074 China

**Keywords:** Silicon nitride slab, Complete photonic bandgap (CPBG), Microcavity, Slab device

## Abstract

The slab effective index difference between the transverse-electric (TE) and transverse-magnetic (TM) polarizations was utilized to obtain complete photonic bandgap (CPBG) in a silicon nitride (Si_*x*_N_*y*_) photonic crystal slab. For this, coincident frequency range in the TE photonic bandgap (PBG) and TM PBG, which denotes the CPBGs of the slab, must be found with the same structure. Through adjusting the effective index pair of TE and TM polarizations by changing the thickness of the Si_*x*_N_*y*_ core layer, and also optimizing the structure parameters within the photonic crystal plane, a large normalized CPBG of 5.62% was theoretically obtained in a slab of Si_*x*_N_*y*_ with a refractive index of 2.5. Moreover, based on the obtained CPBG, a microcavity which could support both TE and TM polarization was theoretically demonstrated. The cavity modes for different polarizations were both well confined, which proved the reliability of the CPBG. In addition, using the same method, the lowest refractive index of Si_*x*_N_*y*_ on silica slab for a CPBG could be extended to as low as 2. The results indicate that there is potential for development of various high-performance CPBG devices based on Si_*x*_N_*y*_ slab technology.

**Graphical Abstract:**

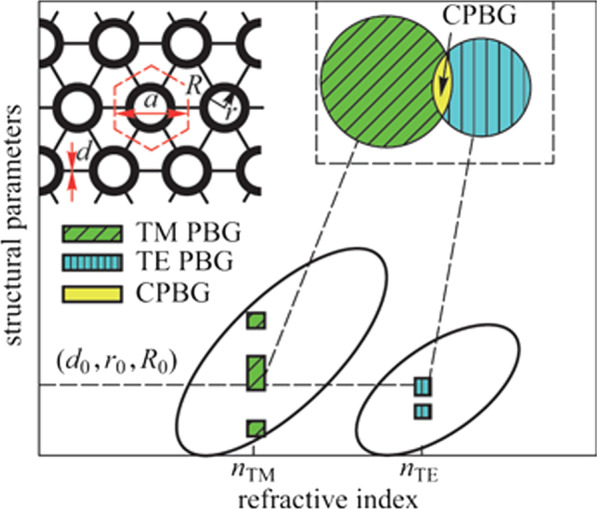

## Introduction

Because of the small mode volumes and high quality factors [1], silicon-based photonic bandgap (PBG) devices have been regarded as the essential elements for miniaturization and integration [2]. Compared with PBG for one single polarization, the complete photonic bandgap (CPBG), which has PBGs for both transverse-electric (TE) and transverse-magnetic (TM) polarizations, is of particular interest for reduced loss of light propagation [3] and the ability to polarize multiplex [4]. Therefore, various powerful silicon photonic devices based on CPBG have been demonstrated theoretically and experimentally, such as high-*Q* microcavity [1], polarization beam splitter [2, 5, 6], polarization-independent waveguide [7, 8], etc. However, currently most of these devices are based on the SOI platform which has a high refractive index contrast (RIC) [2, 3, 5–9]. Unfortunately, bulk crystalline silicon has non-negligible two-photon absorption [10] in all telecommunication bands with wavelengths shorter than about 2000 nm, which seriously affects the efficiency of nonlinear photonic chips in generating and processing all-optical signals [11, 12]. Therefore, some new platforms compatible with CMOS processes, such as silicon nitride (Si_*x*_N_*y*_), Hydex, have been proposed previously [11]. But the RICs of these new material platforms are lower than that of SOI, which makes them difficult to obtain CPBGs. In other words, although the proposal of the new platforms relieves the problem of non-negligible two-photon absorption in crystalline silicon, they cannot inherit the advantage of the easy access to CPBG that is provided by the SOI platform. Therefore, obtaining the CPBG under relatively low RICs corresponding to the new platforms remains a problem.

Many efforts have been made in the past few years to obtain a CPBG at lower RICs [13–15]. By rotation angle, a hexagonal air hole structure based on rutile TiO_2_ with a refractive index of 2.85 has been proposed and a CPBG of 1.5% has been obtained [14]. Moreover, chalcogenide photonic crystal (PC) slow light waveguides with RICs of 2.85 and 2.6 have been reported respectively [15, 16]. Through focused ion beam milling technology, highly efficient evanescent coupling between a chalcogenide glass PC waveguide with RIC of 2.7 and a silica fiber nanowire has been demonstrated [17]. Furthermore, by suitably designed supercell PCs, when the RIC is 2.57 corresponding to diamond and strontium titanate, a hexagonal connecting-rods PC slab has been found to be able to support a CPBG of 5.6% [18]. In summary, CPBGs could potentially be obtained in slabs with moderate RIC material platforms, such as rutile TiO_2_, strontium titanate, diamond and chalcogenide glasses. However, to the best of our knowledge, there is no report about CPBG in a Si_*x*_N_*y*_ slab. The transparent window of Si_*x*_N_*y*_ can be extended from infrared to the visible light band [19]. Moreover, because of the high-quality CMOS-compatible fabrication processes, and of the negligible two-photon absorption at telecommunication wavelengths, Si_*x*_N_*y*_ platform technology is particularly promising [12]. Since the *x* and *y* parameters in Si_*x*_N_*y*_ are adjustable, the refractive index of Si_*x*_N_*y*_ also can be varied between 2 and 3.1 [11, 20, 21]. Therefore, in this work, CPBG in a PC slab with the Si_*x*_N_*y*_ index within 2–2.5 was engineered, making use of the slab effective index [22–24] difference between TE polarization and TM polarization, and which could be adjusted by the slab thickness.

## Scheme and structure

**Fig. 1:**
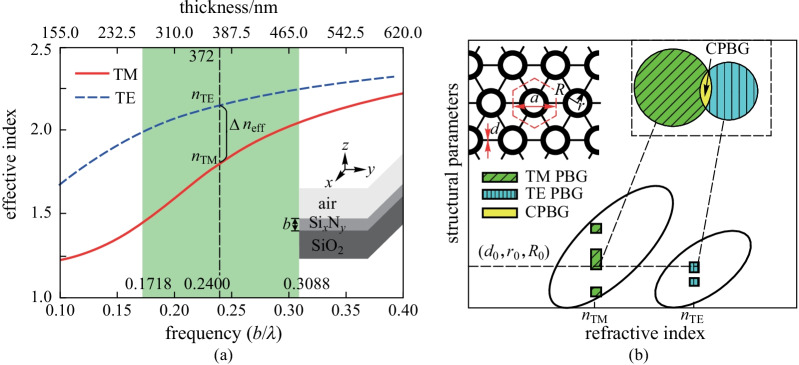
**a** Effective indices of TE and TM polarizations with different frequencies in a Si_*x*_N_*y*_ slab, green shadow denotes the single-mode range, and the right lower inset shows a schematic structure of the slab, in which *b* represents the thickness of the Si_*x*_N_*y*_ guided layer. The lower axis represents the frequency, and the upper axis represents the corresponding thickness of the Si_*x*_N_*y*_ guided layer at the fixed wavelength of 1.55 μm. **b** Schematic diagram of obtaining CPBG in a Si_*x*_N_*y*_ PC slab by utilizing the effective index difference between polarizations. The left upper inset shows a schematic structure of the connecting annular holes PC (CAPC) proposed in this paper, in which the black color represents Si_*x*_N_*y*_ and the white color represents air

In 2D ideal PCs, the lowest RIC for TM PBG is usually lower than that for TE PBG; the lowest RIC for a CPBG is therefore restricted by that for a TE PBG. Accordingly, it has been widely thought that, when the slab effective index is lower than the lowest RIC for a TE PBG of its corresponding 2D type, it is difficult to obtain CPBG in a slab type PC. Fortunately, in a sandwiched slab structure like Si_*x*_N_*y*_ on silica cladding, the slab effective index for TM polarization is usually lower than that for TE polarization, as shown in Fig. 1a. Thus, utilizing the lower slab effective index of TM polarization, and also considering the lowest RIC for TM PBG in 2D ideal PCs is lower than that for TE PBG [18, 25], it is possible to have a TM PBG with an effective index lower than that for a TE polarization. Therefore, if the coincident frequency range in the TE PBGs and TM PBGs could be found with the same structure parameters and with their corresponding effective indices for the two polarizations, obtaining CPBGs of a slab with effective index for TM polarization lower than that for TE polarization is possible, as illustrated in Fig. 1b.

The detail of the scheme to obtain CPBG in a Si_*x*_N_*y*_ PC slab by utilizing the effective index difference between polarizations is shown in Fig. 1. In Fig. 1a, a typical example of the effective indices for the lowest two guided polarization modes in a Si_*x*_N_*y*_ slab is shown. The right lower inset of Fig. 1a shows a schematic structure of Si_*x*_N_*y*_ slab, in which a Si_*x*_N_*y*_ guided layer with a refractive index of 2.5 is sandwiched between a silica sub-cladding layer and an air upper-cladding layer. The thickness of the Si_*x*_N_*y*_ guided layer (represented by *b*), corresponding to each frequency at 1.55 μm wavelength, is also shown. Here, the effective indices for the lowest two guided polarization modes in a Si_*x*_N_*y*_ slab were obtained by considering the light confinement in a vertical dimension (*z* direction) with a 1D method [23]. The effective indices and the photonic band structures in this paper were all calculated with the MPB software developed by MIT, which casts the Maxwell equations as a Hermitian eigenvalue problem with the plane wave expansion technique [26].

In Fig. 1a, one may easily find that the effective index of TM polarization (denoted as *n*_TM_) is lower than that of TE polarization (denoted as *n*_TE_) at the same wavelength or frequency. And at the same wavelength or frequency, the *n*_TE_ and *n*_TM_ are a pair, corresponding to each other. Between normalized frequencies 0.1718 and 0.3088 (*b*/*λ*), we confirmed that both TE and TM polarizations are single mode. It means that within the single mode frequency range denoted as green, there is usually a nonzero Δ*n*_eff_ between *n*_TE_ and *n*_TM_. Meanwhile, as shown in Fig. 1b, using the corresponding pair of effective indices found in Fig. 1a, TM PBGs and TE PBGs can be obtained with different structural parameter ranges represented by the green slash areas and the blue vertical line areas, respectively. Thus, if the overlapped frequency range of the TE PBGs and TM PBGs can be found under the same structural parameter, marked as (*d*_0_, *r*_0_, *R*_0_), CPBG in a PC slab can be obtained, as shown in the yellow area of Fig. 1b. In other words, CPBG in a Si_*x*_N_*y*_ PC slab with low RIC can be obtained.

The key point for obtaining the CPBGs becomes whether we could find the overlap region of TE and TM PBGs under the same structural parameters. To test this, a conventional triangular lattice PC slab was tried first, but the CPBG could not be obtained when the refractive index of the guided layer was lower than 3.5. Therefore, the choice of PC structure is important for obtaining CPBG in the lower RIC. Previous investigations have shown that both annular hole PCs [27, 28] and connecting-rods PCs [29–33] can increase the width of the 2D CPBGs. So here, a new PC formed by connecting annular holes (denoted as CAPC), as shown in the upper left corner of Fig. 1b, was proposed to inherit the features of previous two PCs. In Fig. 1b, *r* and *R* are the inner and outer radii of the annular hole, *d* represents the width of the connecting-rods, *a* is the lattice constant. Since CAPC has more adjustable parameters and greater degrees of freedom, the possibility of obtaining CPBGs should also be greater.

## Optimization for complete photonic bandgap

**Fig. 2:**
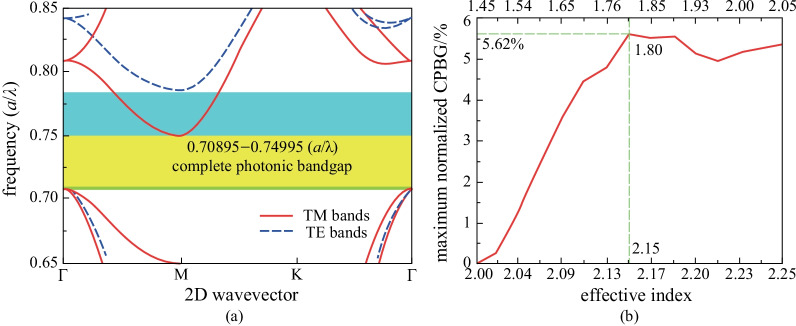
**a** Band diagram of CAPC slab when *n*_TE_ = 2.15 and *n*_TM_ = 1.8, the corresponding structural parameters are *d* = 0.26*a*, *r* = 0.3*a*, and *R* = 0.4*a*. The yellow shadow and blue shadow together denote the PBG for TE polarization (0.708953–0.785195 (*a*/*λ*)), the yellow shadow and green shadow together denote the PBG for TM polarization (0.707918–0.749946 (*a*/*λ*)), and the yellow shadow denotes the 2D slab PC CPBG (0.70895–0.74995 (*a*/*λ*)). **b** Maximum normalized CPBG of CAPC slab with different effective index pairs in the single-mode range, the lower axis represents the *n*_TE_, and the upper axis represents the *n*_TM_

In this stage of the work, optimization of structural parameters was performed to achieve a CPBG in the Si_*x*_N_*y*_ CAPC slab. Firstly, as shown in Fig. 2b, within the whole single-mode frequency range, the effective index pairs (*n*_TE_, *n*_TM_) could be varied by changing the parameters *b* or *λ*. To obtain the maximum normalized CPBG, effective index pairs between single mode normalized frequencies 0.1718 and 0.3088 (*b*/*λ*) with a step size of 0.01 were chosen. Then, at each specific pair of *n*_TE_ and *n*_TM_, the PBGs of TE and TM polarizations in the CAPC were calculated, respectively. Therefore, optimized parameters of planar structure in CAPC could be obtained. During the optimization, each of the three key structural parameters (*d*, *r*, *R*) was first scanned with a step of 0.01*a*, 0.025*a*, and 0.025*a* respectively, to obtain a preliminary view of the coincident frequency range of TE PBGs and TM PBGs under the same parameters. Then the parameter step of *R* and *r* was reduced to 0.005*a* for the structural parameter zones where CPBGs were located. Finally, as shown in Fig. 2a, when *n*_TE_ = 2.15 and *n*_TM_ = 1.8, the largest normalized CPBG of 5.62% was obtained for *d* = 0.26*a*, *r* = 0.3*a*, and *R* = 0.4*a*.

Figure 2b shows the maximum normalized CPBG of the CAPC slab with different effective index pairs in the single-mode range. It can be clearly seen from Fig. 2b that, when *n*_TE_ within the range of 2.13–2.25 (corresponding to *n*_TM_ within the range of 1.76–2.05), relatively large CPBG could be maintained. However, when the *n*_TE_ is lower than 2.09 and *n*_TM_ is lower than 1.65, the size of CPBG begins to decrease sharply. Finally, when *n*_TE_ = 2 and *n*_TM_ = 1.45, CPBG disappears. The results indicate that CPBG is relatively stable within a certain wavelength or frequency range. Thus, in the whole single-mode frequency range, the largest normalized CPBG of 5.62% with *n*_TE_ = 2.15 and *n*_TM_ = 1.8 can be obtained. Moreover, as shown in Fig. 1a, for a wavelength of 1.55 μm, each frequency corresponds to a different thickness of the Si_*x*_N_*y*_ slab. That means, in this frequency range where larger CPBGs are located, we can intentionally choose the Si_*x*_N_*y*_ slab thickness within the fabrication limits. Considering for 1.55 μm wavelength, the structure parameters of the optimized CAPC slab with the largest normalized CPBG of 5.62% can all be designed: *b* = 372 nm, *d* = 293.96 nm, *r* = 339.2 nm, and *R* = 452.2 nm, which should be possible to fabricate using state of the art technology [34, 35].

## Microcavity structure based on the complete photonic bandgap

**Fig. 3:**
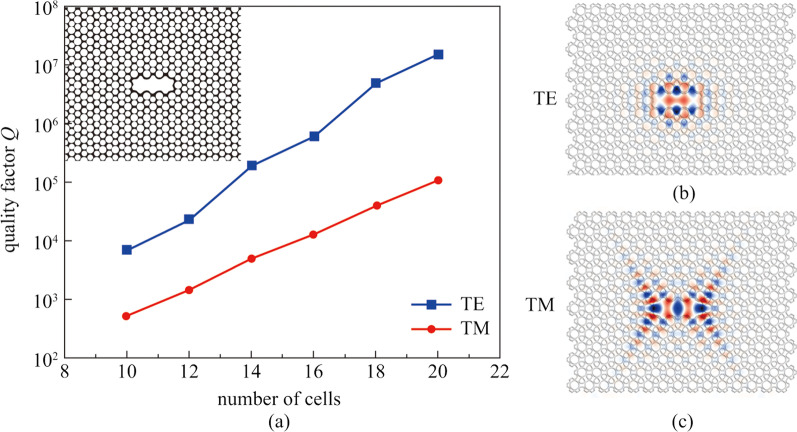
**a** Quality factor of the CAPC cavity, the left upper shows schematic structures of microcavity with cells of 14. **b** *H*_*Z*_ field for TE cavity mode having a *Q* value of 197616 with 14 cells, the *z* direction is shown in the coordinate system in the lower right corner of Fig. 1a. **c** *E*_*Z*_ field for TM cavity mode having a *Q* value of 4968 with 14 cells, the *z* direction is shown in the coordinate system in the lower right corner of Fig. 1a

To further confirm how reliable the CPBG obtained in the triangular-lattice CAPC slab could be, as an example a dual-polarization microcavity was theoretically demonstrated by directly making use of the optimized CAPC slab with *n*(Si_*x*_N_*y*_) = 2.5. We considered removing three CAPC in the middle to form a microcavity for the sake of simplicity, as shown in the upper left illustration in Fig. 3a. To study the light confinement of the microcavity, the effective index model which is the same as in Sect. 2, was considered. It should be clear that the microcavity could support both TE and TM modes. Figure 3a shows the quality factors of the resonance cavity modes as a function of the number of cells surrounding the defect, and when the number of cells is 14, the *Q* values of 197616 and 4968 have been obtained in the CAPC cavity for TE and TM resonance modes, respectively. Figure 3b shows the *H*_*Z*_ field for TE cavity mode having a *Q* value of 197616 with 14 cells, and Fig. 3c shows the *E*_*Z*_ field for TM cavity mode having a *Q* value of 4968 with 14 cells. Actually, during our investigation, we varied the number of the cells, from 10 to 20 with steps of 2. As shown in Fig. 3a, when increasing the number of cells, the *Q* value hugely increases due to existing CPBG of CAPC. This behavior has been explained in a photonic crystal book written by MIT [36]. In our investigation, the *Q* values were obtained by using the Harminv command in the MEEP software [37], accompanied by running very narrow-bandwidth point sources.

## CPBG for Si_*x*_N_*y*_ slab with other refractive indices

After the CPBG of CAPC slab with the RIC of 2.5 had been investigated, another question arose: could it be further extended to a Si_*x*_N_*y*_ slab with lower RIC? Therefore, based on the same method, we also calculated the CPBG of CAPC slab for Si_*x*_N_*y*_ guided layer with other lower refractive indices.

**Fig. 4:**
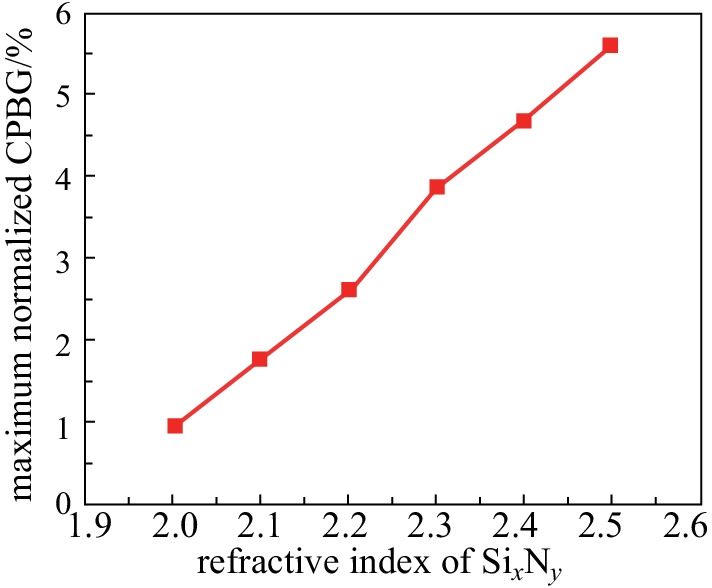
Maximum normalized CPBG of CAPC slab with different refractive indices of Si_*x*_N_*y*_

It can be seen from Fig. 4 that as the refractive index of Si_*x*_N_*y*_ decreases, the CPBG gradually becomes smaller. In other words, the size of the CPBG has a lot to do with the RIC of the material platform. More specifically, the higher the RIC of the material platform, the easier access is to CPBG, which leads to the higher possibility of TE PBGs and TM PBGs overlapping. Actually, the lowest refractive index of Si_*x*_N_*y*_ for a CPBG can be extended to as low as 2. At this time, a normalized CPBG of 0.96% with *n*_TE_ = 1.8 and *n*_TM_ = 1.67 can be obtained, the corresponding structural parameters are *d* = 0.21*a*, *r* = 0.25*a*, *R* = 0.375*a*.

## Conclusions

In conclusion, by utilizing the difference between effective index of TE polarization and TM polarization, large normalized CPBG of 5.62% can be obtained in the 2D Si_*x*_N_*y*_ slab consisting of triangular-lattice CAPC. The investigation indicates that both the method of using effective index difference between TE and TM polarizations and the structure of CAPC are essential for obtaining a large CPBG in the lower RIC. The method is a prerequisite or offers a shortcut for engineering a suitable PC structure to obtain a large CPBG. However, if the method is not compatible with a good performance structure, the effect may not be beneficial. Therefore, the adoption of CAPC is also crucial.

Moreover, a microcavity which could support both TE and TM polarization in the CAPC slab was theoretically demonstrated by making use of the optimized CPBG. Both *H*_*Z*_ field and *E*_*Z*_ field could show that the microcavity has good performance in confining light, which demonstrated the reliability of the CPBG obtained in the triangular-lattice CAPC slab. Based on our study as reported in this paper, other useful applications like polarization-independent waveguide devices can also be suitably designed. Furthermore, the CPBG of CAPC slab for Si_*x*_N_*y*_ with other refractive indices was also performed. We noticed that the CPBG of the Si_*x*_N_*y*_ slab could remain existing until a record low index contrast of 2 in this work. The result indicates that development of high-performance CPBG devices in Si_*x*_N_*y*_ slab would be possible. It also provides encouragement that there may be significant improvements remaining to be discovered in designing and optimizing silicon based CPBG devices.
